# Type 1 Diabetes in Autoimmune Polyendocrinopathy-Candidiasis-Ectodermal Dystrophy Syndrome (APECED): A “Rare” Manifestation in a “Rare” Disease

**DOI:** 10.3390/ijms17071106

**Published:** 2016-07-12

**Authors:** Alessandra Fierabracci

**Affiliations:** Infectivology and Clinical Trials Area, Children’s Hospital Bambino Gesù, Rome 00146, Italy; alessandra.fierabracci@opbg.net; Tel.: +39-06-6859-2656

**Keywords:** Type 1 diabetes, autoimmune polyendocrinopathy-candidiasis-ectodermal dystrophy syndrome (APECED), immunologic features, genetics, etiopathogenesis

## Abstract

Type 1 autoimmune polyglandular syndrome (APS1) is a rare autosomal recessive disease, caused by mutations in the autoimmune regulator gene (*AIRE*); the encoded Aire protein plays an important role in the establishment of the immunological tolerance acting as a transcriptional regulator of the expression of organ-specific antigens within the thymus in perinatal age. While a high prevalence for this rare syndrome is reported in Finland and Scandinavia (Norway), autoimmune polyendocrinopathy-candidiasis-ectodermal dystrophy syndrome (APECED) cohorts of patients are also detected in continental Italy and Sardinia, among Iranian Jews, as well as in other countries. The syndrome is diagnosed when patients present at least two out of the three fundamental disorders including chronic mucocutaneous candidiasis, hypoparathyroidism, and Addison’s disease. Among the associated conditions insulin-dependent diabetes mellitus (Type 1 diabetes) has been rarely reported in different series of patients and occurring more frequently in Finnish APECED patients. In this review, we analyze the incidence of Type 1 diabetes as a clinical manifestation of APECED in different populations highlighting the peculiar genetic and immunological features of the disease when occurring in the context of this syndrome.

## 1. Introduction

Autoimmune polyendocrinopathy-candidiasis-ectodermal dystrophy syndrome (APECED) (OMIM ID: 240300) is an inherited rare autosomal recessive disorder caused by mutations of the *AIRE* (autoimmune regulator) gene (reviewed in [1]). The disease, first reported by Leonard in 1929 [2], is also known as type 1 autoimmune polyglandular syndrome (APS1) or Whitaker’s syndrome. Although rare, a high prevalence for this syndrome is reported in Scandinavian countries, especially in Finland [3]. Cohorts of APECED patients were also reported in continental Italy [4] and Sardinia (1:14,000) [5], among Iranian Jews (1:9000) [6], as well as in other countries [7,8,9]. Clinically, this syndrome is an association of immunodeficiency and autoimmune endocrine deficiencies (reviewed in [3]). The clinical diagnosis requires the presence of at least two of the three main disorders: chronic mucocutaneous candidiasis (CMC), hypoparathyroidism, and primary adrenal insufficiency (Addison’s disease). As a secondary manifestation, other endocrine or non-endocrine autoimmune disorders can be associated (vide infra).

The locus responsible for APS1, the *AIRE* gene has been mapped on chromosome 21q22.3 [2] and since its discovery APECED has represented a pivotal model to understand the mechanisms of immunological tolerance and its loss in the context of autoimmunity. Indeed, the translated Aire protein, whose function is affected by *AIRE* mutations is involved in the transcriptional regulation of the expression of organ-specific antigens within the thymus and the regulation of the negative selection of autoreactive T cell clones [10].

To date over 100 mutations of the *AIRE* gene have been discovered and confirmed the clinical diagnosis of APS1 in different groups of patients together with the testing of anti-interferon ω (IFN ω) autoantibodies (Abs) [10]. Peculiar genotypes were reported in certain populations, thus suggesting a potential founder effect (Table 1) [1,11,12,13,14,15,16,17,18,19,20,21,22].

The discovered mutations are scattered throughout the protein sequence; therefore, few insights into the function of Aire have been achieved from their functional investigation. No specific correlation has been identified so far between a known genotype and a peculiar phenotype within this monogenic syndrome by epidemiological investigation in different populations. The only missense mutation 6 c.682T>G (p.Gly228Trp) was reported in an Italian family with APECED, frequently associated with autoimmune hypothyroidism [23].

In this review we first revise the current knowledge on APECED and especially focus on the incidence of Type 1 diabetes (T1D) as its clinical manifestation in different populations, as well as the peculiar genetic and immunological features of the disorder when developed in the context of the APS1 syndrome.

## 2. Immunological Features of APECED

Among the immunological abnormalities detected in APS1 patients is the presence of autoantibodies (Abs) against affected organs, i.e., Abs against the steroidogenic enzymes P450scc (cytochrome P450 side chain cleavage enzyme), P450c17 (steroid 17a-hydroxylase/17,20 lyase), and P450c21 (steroid 21-hydroxylase) in patients with Addison’s disease and/or gonadal failure [24], diabetes-related Abs specificities including insulin, proinsulin, glutamic acid decarboxylase isoform 65 (GAD65), aromatic l-amino acid decarboxylase (AADC) (vide infra), the insulinoma-associated antigen/tyrosine-phosphatase-like molecule (IA-2), IA-2β or phogrin, thyroid-related Abs, anti-thyroglobulin (Tg) and anti-thyroperoxidase (TPO), celiac disease-related anti-transglutaminase (rTg) Abs, autoimmune enteropathy-related specificities against tryptophan hydroxylase (TPH), and parathyroid autoantigen NACHT (acronym for NAIP (neuronal apoptosis inhibitor protein), C2TA (MHC class 2 transcription activator), HET-E (incompatibility locus protein from *Podospora anserina*), TP1 (telomerase-associated protein)) leucine-rich-repeat protein 5 (NALP5) [25]. Another immunological feature is the presence of circulating neutralizing antibodies to cytokines in the peripheral blood of APECED patients. These specificities are rare in healthy controls and can block class I interferon secretion in vivo and in vitro [26]. Neutralizing Abs to Th (T helper)17 cytokines, i.e., IL-17A, IL-17F, and IL-22, are also present in the peripheral blood of patients causing defective antifungal response, as putative contributors to the occurrence of CMC in APECED [27].

The fundamental pathogenetic mechanisms in APECED appear to be T cell-mediated. Regarding the T cell phenotype, CD8^+^ effector T cells were reported to circulate at increased frequency [28]. Contradictory reports refer to the CD4^+^ T cell population, with Perniola et al. [29] observing an increased frequency of CD4^+^CD25^+^ lymphocytes, while Wolff et al. [30] recognized no differences in the frequency of CD4^+^ activated/memory subtypes among APECED patients and controls. Studies that are more convincing report a defect of the Treg population [31,32]. In particular Kekalainen et al. [32] observed a reduction of the FoxP3^+^ Treg population.

Few authors have investigated the B cell subsets phenotype in APECED patients. It is not known to what extent they are pathogenic, since self-reactive B cells may be important players of the disease process or simply act as bystanders [33]. Mainly, organ-specific autoantibodies are directed against enzymes that play a role in hormonogenesis within the intracellular compartment [34]. However, of note, the production of antibodies is critical in the pathogenesis of autoimmune conditions, such as arthritis, lupus nephritis, oophoritis, and pemphigus [35,36,37,38]. Among the other functions through which B cells can contribute to autoimmune pathology is the secretion of immunomodulatory cytokines [39], the control of lymphoid tissue neogenesis, and lymphangiogenesis [40].

B cells can also exert an effect on dendritic cells (DCs) and regulate T cells. B lymphocytes are capable of regulating the activation status and the number of effector T cells since in B cell-deficient mice abnormal CD4^+^ T cell responses were observed [41,42]. B lymphocytes can act as antigen-presenting cells (APCs); by binding antigens with high affinity they can be more effective than DCs and macrophages at presenting rare antigens [43,44]. The hypothesis of the pathogenic role of B cells in promoting autoimmunity through T cell priming and antigen presentation was also addressed using the non-obese diabetic (NOD) mouse model [45,46]. Proof of evidence regarding the contribution of B cells to multi-organ autoimmunity in *Aire*^−/−^ mice was reported by Gavanescu et al. [33] opening perspectives on the utility of an anti-B cell targeted therapy for the treatment of APECED patients.

A study conducted on 15- to 24-months old *Aire*^−/−^ mice [47] demonstrated that the genetic defect has early consequences in the hematologic development of the monocyte lineage and produces an increased risk to develop marginal zone B cell lymphoma (MZL) with increasing age. Furthermore, liver infiltrates of B cells were also found, suggesting chronic antigen exposure and an exaggerated lymphocyte activation. An increased number of monocytes and metallophilic macrophages in the spleen also demonstrated the effect of Aire deficiency in peripheral tolerance mechanisms.

## 3. Clinical Manifestations of APECED

On a clinical point of view, APECED patients may be affected by a variety of disease components with different degrees of severity and at different time lapses from the appearance of the first manifestation [48]. In the same individual, the number of disease components can vary from 0–9. Furthermore, the onset of the first disease can occur from the age of a few months until adulthood [48]. Generally, patients develop CMC in early infancy or during childhood [49] with the only R257X genotype reported with frequent association with candidiasis. The sequence of appearance of the various diseases may differ in different patients and variations may even occur within the same family. This envisages the potential contribution of other genetic/epigenetic factors other than specific *AIRE* mutations in the disease pathogenesis.

CMC is usually the first symptom. However, this immunodeficiency has a prevalence of 100% in Finnish APECED patients, while in Iranian Jews it is rarely reported [6]. Its severity in patients can vary from signs of inflammation at the corners of the mouth or extended to the whole oral cavity. Candidiasis causing chronic inflammation may also increase the risk for oral carcinoma [50].

Usually the follow-up of APECED patients show the development of autoimmune hypoparathyroidism and Addison’s disease. Hypoparathyroidism is the most common autoimmune component in APECED. Indeed, idiopathic hypoparathyroidism always requires the differential diagnosis with APECED. Addison’s disease is the second most frequent autoimmune disorder reported in 72% of Finnish APECED patients [3]. Especially in females, gonadal failure is a common autoimmune components. During puberty, it is mainly females who develop hypogonadism, specifically during their teen years or in young adulthood. Ovarian insufficiency was reported as the third most frequent autoimmune component, even documented in up to 65% of APECED women [51,52]. It can manifest as primary amenorrhea with failure of arrested pubertal development. In 50% of these female APECED patients premature menopause can occur [51]. Insulin-dependent diabetes (Type 1 diabetes, T1D), alopecia areata, vitiligo, ectodermal dystrophy, non-infectious nail dysplasia, enamel dysplasia, transient skin rash during fever episodes, ocular symptoms (keratoconjunctivitis, dry eye, iridocyclitis, cataract, retinal detachment, and optic atrophy) [53] are other rare associated diseases. APECED patients may also develop autoimmune enteropathies, such as celiac disease, and other intestinal dysfunctions, including chronic diarrhea, constipation, and malabsorption; chronic atrophic gastritis, with or without pernicious anemia (Biermer’s disease), and chronic active hepatitis may also be part of the syndrome. Rarely, APS1 patients may develop tubule-interstitial nephritis or organized pneumonitis and autoimmune thyroid disease [49]. Hyposplenism/asplenia, probably due to an autoimmune mechanism, may occur requiring vaccination against *Streptococcus pneumonia*, *Haemophilus influenza*, *Hepatitis B*, and antibiotic prophylaxis [49].

Some population-based differences were reported for peculiar phenotypic manifestations. For example in Iranian Jew patients, candidiasis and Addison’s disease are less common, whereas in Finnish patients diabetes mellitus is more common than in other ethnic groups (vide supra) [6,54].

## 4. Insulin-Dependent Diabetes Mellitus as a Clinical Manifestation of APECED

T1D is a multifactorial autoimmune disease with a strong genetic component. The disorder occurs in human leukocyte antigen (HLA) genetically-predisposed individuals as a consequence of organ-specific immune destruction of the insulin-producing β cells in the islets of Langerhans within the pancreas [55]. As a genetic background, dominant loci within the major histocompatibility complex (MHC) and the HLA region predisposing to the disease were recognized in both the NOD and human disease. In both Europeans and North Americans of European ancestry susceptibility loci within the HLA complex DRB1 0401, DRB1 0402, DRB1 0405, DQA1 0301, DQB1 0302, or DQB1 0201 alleles were identified. Oppositely, certain HLA alleles DRB1 1501, 1401, or 0701, and DQB1 0602, 0503, or 0303 alleles confer strong protection [55].

The pathogenesis of this multifactorial disorder requires the contribution of genetics, still unknown environmental factors, and stochastic events [55]. It is generally recognized that T1D derives from a breakdown in immune regulation that leads to the expansion of autoreactive CD4^+^ and CD8^+^ T cells, autoantibody-producing B lymphocytes, and activation of the innate immune system.

The rate of T1D incidence varies from 1%–18% of cases in different series of APECED patients [2,3,54,56]. As outlined above, the prevalence of T1D in Finnish APECED patients is considerably elevated [3,54,57] with respect to what is estimated in APECED patients of Eastern and Central European origin [58], Irish populations [16], or Arab families [7] (Table 1), suggesting the influence of genetic factors predisposing to T1D incidence in APECED (see below).

Meloni et al. [1] carried out a prospective investigation on 22 pediatric Sardinian APECED patients. Patients showed severe phenotype with, on average, seven disease manifestations. In addition to the classical triad components, autoimmune hepatitis occurred in 27% of cases with higher incidence in females (5:1). Only one patient developed T1D, and hypothyroidism was not present.

### 4.1. Genetic Factors Predisposing to T1D in APECED Patients

APECED may occur sporadically or among siblings [2]. In unraveling factors that may predispose APECED patients to develop peculiar immunological disorders we have to point out that in early studies no association was reported between APECED and the human leukocyte antigens (HLA) class I or class II [2,49,59]. Subsequently the HLA-A28 haplotype was more frequent in APECED patients than in healthy controls [2,60]. In the same study, the HLA-A3 is shown to be more frequent in APECED patients with associated ovarian failure. Significant differences were not found by examining the HLA class I antigens in 17 patients than in normal controls, while HLA class II (DR genes) were at increased frequency of DR3 and DR5 [2].

More recently with the discovery that APECED is a monogenic disorder due to mutations in the *AIRE* gene, the involvement of allelic variants of the *AIRE* gene in autoimmunity in general was postulated [10]. Since T1D is at high incidence in APECED patients from Finland, *AIRE* variants were suspected to underlie the pathogenesis of T1D in general. *AIRE* gene variants, especially in heterozygosity or sequence polymorphisms, were suspected to influence the development of certain organ-specific autoimmune disorders by affecting the establishment of tolerance at the thymus level in perinatal age [61]. In addition, elevated levels of immunoglobulins class A (IgA) and activated T lymphocytes, showing immunological dysregulation, were detected in the peripheral blood of parents of APECED patients harboring heterozygous *AIRE* mutations [62]. Oftedal et al. [63] recently reported mono-allelic mutations of the *AIRE* gene in multiple cases and families; these variations, located within the first plant homeodomain (PHD1) zinc finger segregated with dominant inheritance. In comparison with classical APECED these variants caused milder phenotypic features at later onset, with reduced penetrance. As opposed to recessive mutations located within the caspase recruitment domain (CARD domain), the same variants had a dominant-negative suppressive effect on gene expression from wild-type *AIRE* [63]. This investigation further demonstrates that AIRE mutations are associated with common organ-specific autoimmunity with a variable phenotype ranging from classical APS-1 to a non-classical form that mimics common organ-specific autoimmunity [63].

The insulin (*INS*) gene is associated with T1D incidence [64]. This association was more probably reported as due to the locus insulin-dependent diabetes mellitus 2 (IDDM2), mapped within the upstream region of the *INS* gene and corresponding to a minisatellite polymorphism of the variable number of tandem repeats (VNTR) of the chromosome 11p15.5 [65]. VNTR plays a role in the ectopic expression of insulin in the thymus, thus inducing susceptibility to T1D [66,67].

The potential interaction of *AIRE* and *INS* genes in the development of T1D was originally unraveled in Finnish APECED patients [65]. They examined a series of 733 Finnish patients and 735 controls and failed to detect an association of any of the five common *AIRE* SNPs selected from the public database (single nucleotide polymorphism database (dbSNP)) (rs2776377, rs878081, rs1800520, rs933150, and rs1800522) or the corresponding *AIRE* haplotypes. The 23*Hph*I polymorphism in the *INS* gene was significantly associated with T1D in the Finnish population (*p* = 6.8 × 10^−12^) [64].

Still in Finland Paquette et al. [68] conducted a subsequent genetic investigation to ascertain the risk of autoimmune diabetes among APECED patients, where the cases with diabetes are more frequently found than in other ethnic groups (vide supra) [69] (Table 2). IDDM, located within the MHC class II region of chromosome 6p21.31, is the most important susceptibility locus for T1D. Protective alleles for T1D such as DRB1*15 and DQB1*0602 are also protective in APECED [48]. Paquette et al. [68] found that IDDM2 is prevalent in the diabetic Finnish APECED patients versus the nondiabetics, adding evidence that loss of Aire function is not exclusive of T1D-development in APECED. Previously, the same authors examined T1D-discordant APECED siblings, from a French Canadian family, having the same *AIRE* mutation [70]. The T1D-affected sibling was found to possess a 5’ INS VNTR I/I genotype while the unaffected sibling had a I/III genotype suggesting the involvement of this locus in APECED-associated T1D. This hypothesis was confirmed in another pair of APECED siblings discordant for T1D.

Adamson et al. [71] investigated a substantial sample of all the APECED patients in the UK population where APECED prevalence is 2–3 cases per million. Patients were genotyped for the HPhI polymorphism, which is in high linkage disequilibrium with the insulin gene VNTR alleles. This study demonstrated in APECED patients an association between T1D incidence and homozygosity for the T1D susceptibility locus class I/INS VNTR allele (Table 2).

Halonen et al. [48] investigated *AIRE* mutations and human leukocyte antigen class II genotypes in a series of APECED patients from different countries (Table 2). The only genotype-phenotype correlation was found for the R257X mutation and the high frequency of CMC. They discovered that individual HLA class II genotype were associated with Addison’s disease, alopecia, and T1D, therefore affecting the development of APECED phenotype.

The associated alleles were those established for these disorders in the absence of APECED since the DB1*03 allele had strong association with Addison’s disease, the DRB1*04 allele with alopecia while the DRB1*15-DQB1*0602, the major protective haplotype for T1D, was also found protective in APECED patients. No specific allele association was predisposed to diabetes in APECED patients. In contrast with the finding that in isolated diseases peculiar HLA alleles are frequently associated with the presence of circulating autoantibodies, Halonen’s study suggested that HLA alleles do not have a strong influence on autoantibody formation.

### 4.2. Markers for Pancreatic Autoimmunity in T1D

The absence of genetic haplotypes predisposed to the disease [2], but also of predisposed markers to pancreatic autoimmunity, was considered responsible for the rare incidence of T1D in APECED.

Regarding pancreatic autoimmunity, major autoantigens in T1D include insulin, glutamic acid decarboxylase (GAD), isoform 65 and 67 (GAD65, GAD67), IA-2, IA-2β, or phogrin, and proinsulin (PI) [72]. Eighty percent of newly diagnosed T1D patients present circulating GAD65 Abs. GAD67 Abs are less frequently encountered, probably as a result of reactivity against epitopes shared with GAD65 [57,73].

Although clinical T1D rarely occurs in APECED patients, elevated titers of GADA (GAD Abs) and ICA (islet cell Abs) were assayed by Björk et al. [73]. However, APECED patients have peculiar immunological features since Abs react with GAD65 epitopes differently from those of non-APECED T1D patients. This is probably due to the fact that the GAD65 autoantigenic molecule is presented to the immune system through alternative pathogenetic mechanisms. These authors compare the reactivity of anti-GAD-containing sera from recent-onset T1D, stiff-man syndrome (SMS), and APECED patients. All sera immunoprecipitated GAD from [^35^S] methionine-labeled rat islet lysates. Nevertheless, serum antibodies from SMS and APECED patients recognized the human GAD conformation on Western blot, while T1D sera did not. APECED and SMS sera inhibited the GAD enzymatic activity, but not with T1D sera. The authors speculated that GAD antibody specificities are an epiphenomenon of the autoimmune insulitis that does not lead to clinical disease manifestations [73].

A similar study was conducted by Ronkainen et al. [74] with 20 T1D and 20 APECED sera to unravel whether there was a possible difference in the humoral response to GAD65 revealed by the presence of epitope and isotope-specific GAD65 Abs. However, in both categories of patients, GAD65 Abs were found to target both the middle, carboxy-terminal and, less frequently, the amino-terminal region of GAD65 providing different results than in Björk’s study [73]. GAD65 Abs were especially of the IgG1 subclass, and less frequently of the IgG2 and IgG4 subclasses. Only the human IgG2-GAD65 Abs were more frequently associated with T1D.

In the series of patients reported by Betterle et al. [2] only one patient developed T1D; and tested negative for ICA. ICA were detectable in 12 out of 40 non-diabetic sera (30%) especially associated with antibodies to GAD65 (GADA). Nevertheless, 10 out of 15 APECED sera tested positive for 51 kDa protein antibodies (66%) (vide infra) without any correlation with the presence of ICA or GADA. Five patients did not develop clinical T1D for a mean follow-up period of eight years.

Gylling et al. [75] tested GAD65, ICA, IA2 and anti-insulin (IAA) Abs, and human leukocyte antigen II alleles in 60 Finnish APECED patients of whom 12 developed T1D (Table 3). 36% of diabetics for whom prediabetic samples were available had IA2 Abs and 36% had IAA. Out of the 48 nondiabetics none had IAA, only two (4%) had IA2 Abs that persisted in the follow-up without development of clinical T1D. Authors concluded that IA2 or IAA Abs have a low sensitivity (36%), although highly specific with a predictive value of 67% for T1D development in APECED patients. Furthermore, Gylling et al. [75] reported data on human leukocyte antigen II alleles obtained by examining 59 patients including 11 with T1D and eight additional nondiabetic patients. The DQB1*0602 allele that is protective for the development of T1D was absent in diabetics, while present in 15 out of 56 nondiabetic patients, and 24 out of 93 normal controls possessed it.

Still, in Finland Perheentupa et al. [54] investigated 47 patients, among which five were diabetic and only one was ICA-positive in repeated testing (Table 3). In a subsequent study eight out of 47 patients were affected by T1D [76]; six of these tested positive for GAD65 Abs, one for GAD67 Abs, and four for ICA. Two samples tested negative for diabetes-related Abs. Instead, among 39 non-diabetic patients, 16 had circulating GAD65 Abs, 11 had GAD67 Abs, and 11 had ICA; 20 had at least one antibody specificity. There was no difference in the age of appearance of GAD65 in patients who developed T1D or not. In patients who did not develop the T1D antibody positivity persisted for 10.1 years, while those who became diabetics, positively persisted for up to 4.4 years. Peripheral blood lymphocytes (PBMC) showed in vitro proliferative response in 15 out of 44 patients, but only in three out of 28 normal controls. When challenged with GAD65 PBMC, 16 out of 28 patients exhibited an increased secretion of interferon gamma (IFN-γ). Instead, this was observed in 19% of normal controls. Cellular responses and levels of IFN-γ secretion correlated negatively with Abs levels. A parallel tendency was only observed in four patients, indicating a reciprocal control of humoral and cellular immunity [77]. Furthermore, the proliferative response to GAD65 was significantly associated with the HLA diabetes risk genotype HLA DQB1*0201; instead, the humoral response was not associated with a particular allele.

Velloso et al. [78] investigated the autoimmune response against the pancreatic β cells in six APECED patients. All APECED sera immunoprecipitated the 51 kDa antigen from [^35^S]-methionine-labelled rat islet cell lysates (Table 3). This novel autoantigen was not detectable in tissue homogenates of other endocrine and non-endocrine organs. Subsequently the 51 kDa antigen was characterized as AADC [79]. This antigen was found unrelated to GAD, since depletion from the islet lysate with GAD did not affect the amount of the 51 kDa autoantigen [78]. APECED patients did not show clinical T1D or had altered insulin response to glucose challenge testing, suggesting that they have an autoimmune response against the islets, which is different from that causing the classical T1D.

In the study by Husebye et al. [80] the presence of AADC Abs was assessed in APECED patients and in patients affected by T1D. These specificities were detected in 51% of APECED patients, in none of the 138 T1D patients, nor in healthy controls. The effect of AADC specificities in the development of T1D in APECED patients remains to be unraveled.

## 5. Conclusions

A rare disease, also referred to as an orphan disease, is any disorder affecting a small percentage of the population. Most rare diseases recognize a genetic background, and are manifested throughout the entire life of a patient, even if symptoms do not immediately appear. In Europe, a disorder is ‘rare’ when it affects less than 1 in 2000 persons [81]. Rare diseases generally present a wide diversity of clinical symptoms and features that vary not only from disease to disease but also from patient to patient affected by the same disease. Furthermore, even relatively common symptoms can hide underlying rare diseases, making it difficult to obtain a correct diagnosis. Therefore, the scientific community has highlighted the importance of unravelling the pathogenesis of rare diseases in order to improve their diagnosis, prediction of onset, and prevention of clinical manifestations.

As extensively reviewed in this manuscript, these considerations clearly apply to APECED, a rare disease that appears often with a cohort of manifestations. Among the endocrine-associated conditions, even more rare is the incidence of T1D in APECED as reported in Finland, UK, or Sardinian population studies [1,11,14]. The rarity of APECED has generally caused difficulties in recruiting biological samples to unravel the relevant disease-related immunological features and even more for the diabetic APECED population. So far, the different humoral response to GAD65 in the diabetic APECED patients than in the non-APECED diabetics has not been ascertained, such as the relevance of the AADC Abs specificities. No *AIRE* SNP association has been found with T1D in APECED patients as for other diseases, except for the R257X genotype frequently associated with candidiasis. Studies on a limited number of cohorts have pointed out to the involvement of the *INS*-23HphI variant or IDDM2 in diabetic Finnish APECED than in non-diabetics. So far, genetic associations with T1D were those already discovered for T1D in general. As it generally applies to orphan diseases, future investigations will help to predict with greater accuracy the T1D onset and outcome in APECED patients and clarify its occurrence from a pathogenetic viewpoint. The discovery of specific immunological and genetic features by means of the application of high-throughput screening platforms will contribute to unravel this issue. This could potentially improve the strategies for immune monitoring and early therapeutic intervention in order to improve the patients’ quality of life.

## Figures and Tables

**Table 1:** *AIRE* peculiar genotypes in certain populations.

*AIRE* Genotype	Population	Reference
c.769 C>T (p.Arg257X)	Finnish, Northern Italian	Nagamine et al. (1997) [11]; Cervato et al. (2009) [12]
c.254A>G (A374G, p.Tyr85Cys)	Iranian Jewish	Björses et al. (2000) [13]
964del13 (p.Cys322fsX372)	British	Pearce et al. (1998) [14]
964del13 (p.Cys322fsX372)	American	Wang et al. (1998) [15]
c.967_979del (p.L323_L327>SfsX51)	Irish	Collins et al. (2006) [16]
R203X	Sicilian	Meloni et al. (2002) [17]; Valenzise et al. (2012) [18]; Valenzise et al. (2014) [19]
A21V	Campania	Capalbo et al. (2012) [20]
W78R	Apulia	Betterle et al. (2012) [21]; Palma et al. (2013) [22]
c.415C>T (p.Arg139X)	Sardinia	Meloni et al. (2012) [1]

**Table 2:** Main studies referring to genes conferring genetic susceptibility to T1D in APECED patients.

Reference	Number of APECED Patients	Frequency of T1D in APECED Patients	Population	Predisposing to T1D
Paquette et al. (2010) [68]	50	16%	Finnish	IDDM2 5’ INS VNRT
Adamson et al. (2007) [71]	33	24%	UK	class I/INS VNTR susceptibility locus in 75%
Halonen et al. (2002) [48]	104 (index)	12.5%	12 different	DRB1*03, DRB1*04 found in 61.5% of diabetic APECED and 42.9% of non-diabetics

T1D: Type 1 diabetes; APECED: autoimmune polyendocrinopathy-candidiasis-ectodermal dystrophy syndrome; IDDM2: insulin-dependent diabetes mellitus 2; VNRT: variable number of tandem repeats.

**Table 3:** Main studies reporting testing of diabetes-related autoantibodies tested in sera of APECED patients affected by T1D.

Reference	Number of APECED Patients with T1D	ICA	GAD65 Abs	IA-2 Abs	IAA	AADC
Perheentupa et al. (2002) [76]	8	50%	75%	NT	NT	NT
Gylling et al. (2000) [75]	12	NT	NT	36%	36%	NT
Husebye et al. (1997) [80]	9	NT	55.5%	NT	NT	55.5%
Velloso et al. (1994) [78]	6	NT	83.3%	NT	NT	100%

ICA: islet cell Abs; GAD65: glutamic acid decarboxylase isoform 65; Abs: autoantibodies; IA-2: the insulinoma-associated antigen/tyrosine-phosphatase-like molecule; IAA: anti-insulin; AADC: aromatic l-amino acid decarboxylase; NT = not tested.
